# Five-Year Clinical Outcomes of Local versus General Anesthesia Deep Brain Stimulation for Parkinson's Disease

**DOI:** 10.1155/2019/5676345

**Published:** 2019-01-17

**Authors:** Sheng-Tzung Tsai, Tsung-Ying Chen, Sheng-Huang Lin, Shin-Yuan Chen

**Affiliations:** ^1^Department of Neurosurgery, Tzu Chi General Hospital, Tzu Chi University, Hualien, Taiwan; ^2^Institute of Medical Sciences, Tzu Chi University, Hualien, Taiwan; ^3^Department of Anesthesiology, Tzu Chi General Hospital, Tzu Chi University, Hualien, Taiwan; ^4^Department of Neurology, Tzu Chi General Hospital, Tzu Chi University, Hualien, Taiwan

## Abstract

**Background:**

Studies comparing long-term outcomes between general anesthesia (GA) and local anesthesia (LA) for STN-DBS in Parkinson's disease (PD) are lacking. Whether patients who received STN-DBS in GA could get the same benefit without compromising electrophysiological recording is debated.

**Methods:**

We compared five-year outcomes for different anesthetic methods (GA vs LA) during STN-DBS for PD. Thirty-six consecutive PD patients with similar preoperative characteristics, including age, disease duration, and severity, underwent the same surgical procedures except the GA (*n*=22) group with inhalational anesthesia and LA (*n*=14) with local anesthesia during microelectrode recording and intraoperative macrostimulation test. Surgical outcome evaluations included Unified Parkinson's Disease Rating Scale (UPDRS), Mini-Mental Status Examinations, and the Beck Depression Inventory. Stimulation parameters and coordinates of STN targeting were also collected.

**Results:**

Both groups attained similar benefits in UPDRS part III from STN-DBS (GA 43.2 ± 14.1% vs. LA 46.8 ± 13.8% decrease, *p*=0.45; DBS on/Med off vs. DBS off/Med off) and no difference in reduction of levodopa equivalent doses (GA 47.56 ± 18.98% vs. LA 51.37 ± 31.73%, *p*=0.51) at the five-year follow-up. In terms of amplitude, frequency, and pulse width, the stimulation parameters used for DBS were comparable, and the coordinates of preoperative targeting and postoperative electrode tip were similar between two groups. There was no difference in STN recording length as well. Significantly less number of MER tracts in GA was found (*p*=0.04). Adverse effects were similar in both groups.

**Conclusions:**

Our study confirmed that STN localization with microelectrode recording and patient comfort could be achieved based on equal effectiveness and safety of STN-DBS under GA compared with LA.

## 1. Introduction

The efficacy of subthalamic nucleus deep brain stimulation (STN-DBS) for Parkinson's disease (PD) has been well-documented and has become a standard treatment for patients who have suffered medication-related side effects. Given the importance of the electrode position within the STN topography, imaging for targeting and meticulous electrophysiological mapping are the most powerful tools available for neurosurgeons to refine DBS procedures [1, 2]. Adverse effects resulting from STN-DBS are rare, but some may lead to significant disability, such as intracranial hemorrhage. Most functional neurosurgeons still seek ways to optimize this surgical procedure [3].

Detailed electrophysiological microelectrode recordings (MER) in awake patients under local anesthesia (LA) provide the most accurate and original neural characteristics of the STN for localization, which could explain why most centers still prefer electrode implantation under LA, and most patients can withstand the entire process. However, awake DBS procedures may lead to several limitations. First, most patients have to withstand the entire operation off medication. Second, a meta-analysis revealed that implanting DBS under LA caused more incidences of DBS lead passes than with general anesthesia (GA) and more DBS complications, including intracranial hemorrhage [4]. This reduced risk from GA for STN-DBS might be resulted from more accurate intraoperative imaging, stable blood pressure control, and patients' still position [5]. Third, suboptimal placement of electrodes for deep brain structures such as STN might occur from brain shifts with long procedures [6]. Furthermore, more studies have demonstrated that the short-term surgical outcomes of the asleep STN-DBS method achieved equivalent benefits as STN-DBS under LA in other reports [7].

While several reports have compared the outcomes of asleep STN-DBS with other groups using awake techniques, wide variations in imaging, surgical procedures, and short-term outcomes might not fully address the question of identifying the suitable anesthesia for each patient [5]. Our previous comparative cohort study suggested similar short-term outcomes of PD patients with STN-DBS using GA and LA based on the same procedural protocol [8]. With the paucity of long-term outcomes of direct comparisons between different anesthesia techniques, we analyzed our long-term outcomes in this report.

## 2. Methods

### 2.1. Patient Selection

All PD patients who received medical care and underwent STN-DBS surgery at Hualien Tzu Chi General Hospital were included. They met the United Kingdom Parkinson's Disease Brain Bank diagnostic criteria, in which at least two of the cardinal symptoms were present. Before surgery, each patient underwent a levodopa test to ensure a positive levodopa response (Unifed Parkinson's Disease Rating Scale (UPDRS) part III > 30% improvement in scores). Brain MRI was performed preoperatively to rule out structural abnormalities in every patient. The same surgical team with one senior neurosurgeon (Chen) performed all of the DBS procedures for these patients at our hospital. The detailed evaluation and surgical procedures have been described in previous reports [8–10]. Given the lack of level I evidence concerning long-term outcomes of STN-DBS surgeries between GA and LA, the choice of which type of anesthesia during STN-DBS was determined by the patient's preference after we informed the patients and their families about the potential benefits and risks of both the GA and LA methods.

The institutional review board of Tzu Chi General Hospital approved this study (no. 097-08).

### 2.2. Stereotactic Procedures, Anesthesia, and Microelectrode Recording

The images were obtained with a 1.5 tesla MR unit (General Electric). The standard settings consisted of T1-weighted axial images at 0.75 mm thickness, T2-weighted axial images at 2 mm thickness, and T1-weighted images with contrast. Each of these sequences was performed in contiguous slices. The images were transferred to the Digital Image Communications in Medicine database using the picture archiving and communicating system and the Stealth neuronavigation workstation (Medtronic). The image fusion software fused the three sets of MR images. The tentative surgical target coordinates for the tip of the permanent implantable electrode were set at the central border of the STN by direct visualization from MRI and were adjusted according to the relative position of the red nucleus.

A Leksell G frame (Elekta Instrument Inc., Norcross, GA, USA) was used for the stereotactic procedure. The patient rested on a chair with the head frame fixed under LA. Both groups of patients were given preoperative computed tomography (CT) exams for imaging fusion. The target coordinates were applied to the stereotactic frame and the working stage. Patients in the LA group were placed in the supine position, with their heads and frames fixed to the operative table. After a typical MER of the STN with adequate length was obtained, a permanent electrode was implanted.

The GA group patients received general anesthetics with endotracheal intubation. Anesthesia was induced by administration of fentanyl (1-2 *μ*g/kg), propofol (1–2.5 mg/kg), and a muscle relaxant (rocuronium at 0.6–1.5 mg/kg or cisatracurium at 0.15–0.2 mg/kg). Desflurane or sevoflurane inhalation was maintained during the surgical course and used to keep minimal alveolar concentration at 1 during scalp incision and skull hole creation. The depth of the anesthesia was adjusted by reducing minimal alveolar concentration of inhalational anesthetics at 0.5–0.8 during MER. Medications such as propofol or fentanyl which could decrease neuronal characteristics of STN are avoided during surgery [11, 12]. The patient was monitored by heart rate and blood pressure so that they would not experience a cough reflex or any changes in heart rate or blood pressure during the MER procedure [13]. For both the GA and LA groups, passive movement of the contralateral limb was tested during the MER in the STN to observe whether there were any movement-related neuronal firing changes. The selection of the final trajectory for electrode implantation depended on the length of the STN neuronal firings and the presence of passive movement-related activity of the STN neuron.

The signal obtained from the tip of the microelectrode was sent to the intraoperative MER system (Leadpoint, Medtronic, Minneapolis, Minn., USA), where it was magnified and displayed. In the LA group, an intraoperative test stimulation of up to 4 V was performed to test for adverse effects and the immediate effectiveness of the stimulation. We did not perform any intraoperative test stimulations for the GA group.

Brain CT scanning was performed in 1.25 mm consecutive slices after DBS electrode implantation in both groups to exclude intracranial hemorrhage and to evaluate the postoperative electrode coordinates using image fusion with the preoperative MR images [14]. An implantable pulse generator was implanted in the second-stage operation with 1 week of recovery time after electrode implantation.

### 2.3. Outcome Analysis

We conducted follow-up visits with patients at 1 and 5 years after implanting the DBS electrodes. At the time of the follow-up, each patient was evaluated with the UPDRS in four different conditions: on and off medication and with and without DBS. The off-medication condition (Med off) was defined as a patient who had not taken antiparkinsonian medication for at least 12 hours. The without DBS condition (DBS off) was defined as a patient who had not received DBS for 4 hours or for the time of the tolerance of the patient (sometimes less than 4 hours but at least 1 hour).

To evaluate the effectiveness of STN-DBS, we compared the UPDRS scores in patients in the status of postoperative DBS on/Med off with those from the postoperative DBS off/Med off condition. An improvement was defined as the percentage of change in the difference between the UPDRS scores. The cardinal symptoms from the UPDRS part III were grouped for analysis as follows: tremor (items 20 and 21), rigidity (items 22), bradykinesia (items 23, 24, 25, and 26), posture and gait (items 28 and 29), and axial features (items 18, 19, 27, 28, 29, and 30). Neuropsychiatric function was evaluated using the Mini-Mental Status Examination (MMSE), the Cognitive Abilities Screening Instrument (CASI), and the Beck Depression Inventory (BDI).

### 2.4. Statistical Analysis

Statistical analyses were performed using SPSS software (SPSS statistics 12.0; SPSS Inc.). Mann–Whitney *U*-test and the Wilcoxon signed-ranked test were used as nonparametric tests for categorical data and numerical scores comparisons as appropriate. To compare the extent of improvement difference between groups and side effects incidence, the nonparametric test was also used. Significance was set at *p* < 0.05 for all tests.

## 3. Results

From February 2002 to January 2015, 165 consecutive PD patients underwent bilateral STN-DBS. Among them, 144 patients were evaluated at 1 year and 56 completed 5 years of follow-up. As we followed postoperative outcomes by four different clinical conditions to identify effectiveness of medication or DBS, 36 patients fulfilled this evaluation in this study. A total of 22 (15 males and 7 females) patients decided to be enrolled in the GA group and received desflurane or sevoflurane GA with endotracheal intubation during bilateral STN electrodes implantation, and 14 (12 males and 2 females) patients preferred the LA method during surgery and received only regional anesthesia.

There were no remarkable differences in demographics between the GA and LA groups except the age at surgery. Patients in the LA group were younger when they underwent STN-DBS compared with the GA group (*p*=0.04). The disease durations were similar between groups. The mean follow-up times were 60.9 ± 7.9 months in the GA group and 65.4 ± 9.0 months in the LA group. In terms of their preoperative disease severity (disease duration, UPDRS four-part scores, and H&Y staging) and levodopa response, both groups showed similar motor disability before bilateral STN-DBS was performed (Table 1).

At the 5-year follow-up, both the GA and LA groups showed significant improvements from bilateral STN-DBS (Table 2). For UPDRS part III scores (motor disability), STN-DBS improved 43.2 ± 14.1% in the GA group (DBS on/Med off vs DBS off/Med off) and 46.8 ± 13.8% in the LA group. Between-group analysis did not reveal a difference in the effectiveness of STN-DBS. This similar improvement from STN-DBS also was found at the one-year follow-up for both groups (supplementary table available here). In addition, combined effectiveness from both DBS and medication in both groups also reaches similar extent (Table 3). There were also significant reductions in the levodopa equivalent daily dose (mean percentage of dose reduction between GA and LA: 47.56 ± 18.98% and 51.37 ± 31.73%). Cognitive function remained stable at five years after STN-DBS in both groups. For the GA group, mean preoperative MMSE score was from 27.4 ± 2.6 to postoperative 25.5 ± 4.4 and from preoperative 28.3 ± 1.4 to postoperative 26.4 ± 3.4 in the LA group. In terms of amelioration of depression, mean BDI improved 38.8 ± 10.4% in the GA group and 38.2 ± 11.8% in the LA group at the five-year follow-up.

The stimulation parameters of STN-DBS of both groups showed nearly same results with regard to amplitude, pulse width, and rate. The mean stimulation parameters of Channel 1 (Ch1)/Channel 2 (Ch2) in the GA group were voltage (3.7 ± 0.4/3.6 ± 0.6 V), pulse width (63 ± 9/61 ± 6 microseconds), and frequency (123 ± 20/123 ± 20 Hz). In the LA group, the parameters of Ch1/Ch2 were voltages (3.5 ± 0.6/3.4 ± 0.5 V), pulse width (65 ± 11/62 ± 8 microseconds), and frequency (130 ± 15/132 ± 15 Hz). As the accuracies of STN targeting and DBS electrode position are closely related with long-term effectiveness of DBS, we also compared the surgical coordinates of both the preoperative STN targeting and postoperative stimulation contacts between groups. The mean coordinates for preoperative STN targeting were *X* = 10.85 ± 0.64, *Y* = 3.05 ± 0.53, *Z* = 5.70 ± 0.74 in the GA group and *X* = 11.06 ± 0.56, *Y* = 2.85 ± 0.68, *Z* = 5.48 ± 0.62 in the LA group (*p*=0.21). Meanwhile, the coordinates for postoperative STN were *X* = 10.98 ± 0.94, *Y* = 2.97 ± 0.67, *Z* = 5.24 ± 0.59 in the GA group and *X* = 11.21 ± 1.46, *Y* = 2.98 ± 1.18, *Z* = 5.46 ± 0.82 in the LA group (*p*=0.45). There was no difference in recorded STN length (GA 4.76 ± 0.59 vs LA 4.68 ± 0.71 mm, *p*=0.55). However, the number of MER tracts per hemisphere in GA was significantly less than the number in LA (GA 1.91 ± 1.46 vs LA 2.73 ± 1.82, *p*=0.04). The postoperative adverse effects were similar between two groups (Table 4). The data used to support the findings of this study are included within the article.

## 4. Discussion

Our results demonstrate that PD patients with motor disability and drug-related side effects undergoing STN-DBS through either LA or GA strategies could experience significant amelioration of motor disability at the 5-year follow-up. Between-group analysis also revealed similar extents of improvement in UPDRS scores (including subscale scores), LEDD reduction, and stimulation parameters. In terms of surgical-related complications, adverse events did not show significant differences between groups.

With up to 10 years of long-term outcome analysis reported, LA during surgery has been the mainstream treatment modality for most DBS centers to ensure accurate MER recording and intraoperative test stimulation [15–17]. UPDRS part III scores can be markedly improved by STN-DBS under LA, with the extent of improvement ranging from 45% to 50% [16]. Comparing most studies in the literature with the long-term outcomes of the LA group, Fluchere et al. provided a large cohort of PD patients under controlled GA for DBS and demonstrated significant and sustained benefit 5 years after STN-DBS (the improvements in the UPDRS III scores in Med Off/DBS On were 61% at 1 year and 37% at 5 years) [7]. Our single-center report not only showed direct comparisons of consecutive PD patients under standard and the same surgical procedures except for the anesthetic modalities but also confirmed the noninferiority of the GA method.

Misplacement of DBS electrodes is always a major concern for STN-DBS under GA [18, 19]. Successful STN-DBS implantation and clinical outcomes rely on implanting electrodes with electrophysiological refinement and intraoperative macrostimulation testing with the patient's immediate feedback [20]. Both aspects have been claimed as caveats of DBS under sedation or asleep [21]. Our results of similar stimulation parameters, coordinates of electrode tip, and adverse effects indicate comparable surgical accuracy between the two groups. A deviated MER trajectory may lead to more recording time and associated risks. In addition, suboptimal placement of DBS electrodes usually leads to unprecedented adverse effects from stimulation due to current diffusion or higher power consumption. [22] The advancement of imaging modalities and the use of controlled anesthesia may provide neuropsychiatric patients with less uncomfortable surgeries without compromising surgical benefits [11, 18, 23].

In this report, most adverse effects were transient and resolved after the adjustment of stimulation parameters. One patient in the GA group developed intracranial hematoma and hemiparesis after STN-DBS and another patient had asymptomatic intracranial hematoma in the same group. Although there was no hemorrhage in the LA group, the incidence of intracranial hematoma in the GA group was similar to the previously reported complications from either awake or asleep DBS procedures [7, 24]. There were no significant differences with regard to other stimulation adverse effects between the GA and LA groups in our report, a result that could be anticipated because of both groups having equivalent coordinates of active contacts [25–27]. Although we have less instrumented passes of MER in the GA group, relatively higher rates of hypophonia and dysarthria in GA cohort suggest this speech outcomes warrants future research to elucidate variable influence from STN-DBS, anesthesia method, or surgical techniques [5,27–29]. Hypomania after DBS has been reported and seems to be more correlated with STN stimulation, possibly resulting from the add-on effects from stimulation and dopaminergic medication. Although younger age with STN-DBS may correlate with more postoperative psychiatric effects of DBS, this was not found in our LA cohort [26].

MERs could be accurately recorded in PD patients under GA with inhaled anesthetic, with similar features in the neurophysiological analysis of the STN as those in LA [13]. Although studies suggested decreased STN firing characteristics with Propofol, our equivalent recorded STN spans from MER of both groups indicate that inhalational anesthetics would not preclude MER from detecting reliable STN margins [12, 30, 31]. Based upon accurate electrophysiological and radiological guidance, the long-term outcomes of the GA group in our study were comparable in efficacy with the LA group and the references. Although brain imaging, such as intraoperative MRI, may obviate the necessity of MER and its rare associated risks, we believe that a broader knowledge of the neural underpinning of PD neurophysiology could provide a clearer blueprint for optimized stimulation adjustment [18, 32]. Several centers have deciphered neural recordings of STN to predict individualized stimulation parameter combinations before adjusting them based upon the clinicians' variable levels of experience and the patients' trial responses [33]. Without sacrificing the quality of MER, STN-DBS under controlled GA may detect the underlying detailed neurophysiology of the STN and provide an opportunity for neuroscientists to better understand how anesthetics influence neural recordings.

There were some limitations in our study design. First, the present study was a retrospective study, although the patients in the two groups were consecutively recruited and met all follow-up schedules after surgery. Second, we aim at showing the long-term outcome of two groups in the single center, which results into small case numbers from each group. This may undermine the statistical analysis for lack of power and fail to reveal difference. For example, both groups have similar reduction of MMSE (1.9 points decrease at the five-year follow-up), and this does not reach significant reduction compared to preoperative status. However, this cognitive decline is still within acceptable range [34]. Third, patients enrolled in the GA and LA groups were not randomized and selected their anesthesia method after being completely informed about both. When we started the study, there is no available data about clinical outcome of STN-DBS with GA until Hertel et al. published their preliminary outcomes in 2006 [11]. We informed them the potential discomfort during awake microelectrode recording and implantation of STN-DBS for choosing LA, while for those who opt for GA might have higher risks of deviated electrodes and stimulation side effects due to lower quality MER and unavailable macrostimulation test. They all underwent stringent and blinded preoperative evaluations by a movement specialist, which should eliminate part of the selection bias. Interestingly, a recent report showed that more patients would prefer asleep DBS surgery provided that both surgical and anesthetic methods are available [35]. This might explain the discrepancy of patient numbers between both groups in our study. Identifying factors associated with patients' preference of anesthetic techniques and better clinical outcomes may allow more patients to achieve the benefits of DBS without an intimidating surgical experience.

## 5. Conclusion

In our cohort of PD patients receiving the same surgical techniques except for the type of anesthesia, long-term effectiveness, and safety of STN-DBS under GA is similar to surgery under LA. In addition, our finding suggested that patients would prefer the GA technique during STN-DBS implantation if they were informed both anesthetic options. However, future studies incorporate a randomized trial design, and larger case numbers will facilitate identifying factors for patients to consider before choosing either GA or LA methods during STN-DBS.

## Figures and Tables

**Table 1:** Demographics and preoperative disease severity in the general anesthesia (GA) and local anesthesia (LA) groups.

	GA			LA			*P* value
	Mean ± SD			Mean ± SD			
Sex	*F* = 7/M = 15			*F* = 2/M = 12			0.24
Age at onset	47.5 ± 9			39.6 ± 12.9			0.06
Disease duration (years)	10.2 ± 4.3			9.9 ± 6.9			0.34
Age at surgery	57.7 ± 7.4			49.4 ± 12.2			0.04^*∗*^
Follow-up (months)	60.9 ± 7.9			65.4 ± 9			0.14

UPDRS	Levodopa off	Levodopa on	Improvement (%)	Levodopa off	Levodopa on	Improvement (%)	(%)
	Mean ± SD	Mean ± SD	Mean ± SD	Mean ± SD	Mean ± SD	Mean ± SD	

Part I	4.1 ± 2.1	3.2 ± 1.9	25.6 ± 31.8	3.6 ± 1.8	2.5 ± 1.2	22.9 ± 27.9	0.88
Part II	19.5 ± 8	9.5 ± 4.4	48.9 ± 23.7	19.1 ± 9.6	8.2 ± 4.1	52.4 ± 27.5	0.73
Part III	46.3 ± 14.4	24.3 ± 10.9	48.1 ± 16.3	42.9 ± 17.4	23.1 ± 8.5	43.5 ± 17.3	0.35
Brady	19.1 ± 6.4	11.8 ± 5.8	39.7 ± 19.2	18 ± 6.3	10.4 ± 4.1	39.9 ± 18.8	0.75
Tremor	6.4 ± 4.6	1.5 ± 2.8	70.3 ± 37.8	5.4 ± 4.3	1.5 ± 2	63.2 ± 41	0.61
Rigidity	10.2 ± 3.9	4.7 ± 3.3	55.6 ± 27.8	10.1 ± 5.2	5.6 ± 3.8	50.2 ± 24.4	0.5
Posture and gait	3.8 ± 1.4	2.1 ± 1.1	44.3 ± 26.3	4 ± 1.9	2.1 ± 1	46.6 ± 21.8	0.85
Axial	8.8 ± 3.4	5.2 ± 2	38.5 ± 21.3	8.3 ± 3.8	5.1 ± 1.9	35.6 ± 21.5	0.55
Part IV		6.2 ± 4.4			6.2 ± 4.4		1
Total	76 ± 23.8	43.2 ± 16.9	43.2 ± 16.2	71.9 ± 29.8	40 ± 12.6	40.7 ± 15.6	0.52
H&Y stage	3.3 ± 0.8	2.6 ± 0.3	17.9 ± 13.4	3 ± 0.9	2.3 ± 0.4	17.5 ± 17.4	0.69
ADL score	74.5 ± 22	93 ± 6.3	20.4 ± 22.3	77.1 ± 29.7	97.9 ± 4.3	20.9 ± 30.4	0.51

UPDRS: Unified Parkinson's Disease Rating Scale; H&Y: Hoehn and Yahr; ADL: activities of daily living. ^*∗*^ *P* < 0.05.

**Table 2:** Comparison of postoperative outcomes by DBS between general anesthesia (GA) and local anesthesia (LA) groups at 5 years.

	GA			LA			*P* value
	Levodopa off/DBS off	Levodopa off/DBS on	Improvement (%)	Levodopa off/DBS off	Levodopa off/DBS on	Improvement (%)	
UPDRS	Mean ± SD	Mean ± SD	Mean ± SD	Mean ± SD	Mean ± SD	Mean ± SD	(%)
Part I	4.8 ± 2.2	3.1 ± 1.8	36 ± 28.9	5 ± 2	3 ± 1.9	37.6 ± 26.3	0.84
Part II	26.1 ± 8.5	12.9 ± 4.7	47.6 ± 20.2	23.7 ± 9.2	11.9 ± 5.2	45.6 ± 19.2	0.7
Part III	50.4 ± 12.2	28.6 ± 9.3	43.2 ± 14.1	48.1 ± 14.1	24.6 ± 7.8	46.8 ± 13.8	0.52
Brady	20.8 ± 3.9	14.4 ± 4.3	30.7 ± 15.9	20.7 ± 5.9	12.6 ± 3.3	36.2 ± 17.5	0.38
Tremor	7.2 ± 6.3	1.9 ± 2.8	60.7 ± 37.1	5.4 ± 4.5	1.3 ± 1.3	62 ± 40.3	0.72
Rigidity	10.2 ± 3.8	4.6 ± 3.2	55.4 ± 26.6	10.4 ± 4.3	3.6 ± 3.2	64.5 ± 24.5	0.28
Posture and gait	4.3 ± 1.4	3 ± 1.1	30 ± 18.5	4.4 ± 1.4	2.8 ± 1.1	34.5 ± 22.6	0.35
Axial	10.1 ± 3.3	6.6 ± 2.4	33.4 ± 19.2	9.9 ± 4.1	5.9 ± 2.4	34.9 ± 20.7	0.83
Part IV	5 ± 2.4	2.4 ± 1.8	28.2 ± 85.5	5.6 ± 1.4	1.8 ± 1.6	67.4 ± 27	0.07
Total	86.4 ± 21.5	47 ± 12.7	44.5 ± 13.2	82.4 ± 25.2	41.4 ± 13.1	47.7 ± 13.3	0.44
H&Y stage	3.5 ± 0.8	2.6 ± 0.4	23.3 ± 19.6	3.5 ± 1	2.6 ± 0.6	7.6 ± 14.4	0.7
ADL score	56.4 ± 21.5	85.5 ± 10.6	34.1 ± 23.1	56.4 ± 25.9	84.3 ± 15	10.7 ± 27.1	0.92

UPDRS: Unified Parkinson's Disease Rating Scale; H&Y: Hoehn and Yahr; ADL: activities of daily living.

**Table 3:** Comparison of postoperative outcomes following combined DBS and levodopa effectiveness between two groups at 5 years.

	GA			LA			*P* value
	Levodopa off/DBS off	Levodopa on/DBS on	Improvement (%)	Levodopa off/DBS off	Levodopa on/DBS on	Improvement (%)	
UPDRS	Mean ± SD	Mean ± SD	Mean ± SD	Mean ± SD	Mean ± SD	Mean ± SD	(%)
Part I	4.8 ± 2.2	2.9 ± 1.7	39.9 ± 27.7	5 ± 2	2.8 ± 2.1	42.3 ± 28.1	0.66
Part II	26.1 ± 8.5	10.9 ± 4.9	55.7 ± 18.7	23.7 ± 9.2	9.2 ± 6.4	57.4 ± 22.4	0.86
Part III	50.4 ± 12.2	23.5 ± 9	53.5 ± 14.9	48.1 ± 14.1	18.9 ± 7.5	59.1 ± 13.1	0.38
Brady	20.8 ± 3.9	12.5 ± 4.7	40.5 ± 17.6	20.7 ± 5.9	11 ± 3.9	44.7 ± 19.5	0.47
Tremor	7.2 ± 6.3	1 ± 1.6	75.8 ± 33.7	5.4 ± 4.5	0.3 ± 0.6	84.1 ± 35.9	0.1
Rigidity	10.2 ± 3.8	3.2 ± 2.6	70.2 ± 21.8	10.4 ± 4.3	1.5 ± 1.5	84 ± 16.3	0.07
Posture and gait	4.3 ± 1.4	2.6 ± 1.2	40.2 ± 23	4.4 ± 1.4	2.2 ± 1.2	47.6 ± 25.5	0.18
Axial	10.1 ± 3.3	5.8 ± 2.4	42.4 ± 19.2	9.9 ± 4.1	5.1 ± 2.7	44.2 ± 21.6	0.59
Part IV	5 ± 2.4	2.4 ± 1.8	28.2 ± 85.5	5.6 ± 1.4	1.8 ± 1.6	67.4 ± 27	0.07
Total	86.4 ± 21.5	39.6 ± 12.6	53.2 ± 13.3	82.4 ± 25.2	32.7 ± 14.9	58.5 ± 14.2	0.27
H&Y stage	3.5 ± 0.8	2.4 ± 0.4	28.3 ± 17.3	3.5 ± 1	2.6 ± 0.6	22.9 ± 18.3	0.36
ADL score	56.4 ± 21.5	86.8 ± 9.9	35.3 ± 22.8	56.4 ± 25.9	87.9 ± 18.1	37.2 ± 23.9	0.87

UPDRS: Unified Parkinson's Disease Rating Scale; H&Y: Hoehn and Yahr; ADL: activities of daily living; GA: general anesthesia; LA: local anesthesia.

**Table 4:** Comparison of adverse effects between GA and LA groups.

Postoperative morbidity	GA		LA		*P* value
	*N*	(%)	*N*	(%)	
*Mortality*	0	0	0	0	
Adverse effects related to stimulation					
Hypophonia	5	22.7	1	7.1	0.18
Increased libido	0	0	2	14.3	0.16
Decreased memory	3	13.6	1	7.1	0.55
Paresthesia	1	4.5	0	0	0.43
Dyskinesia	2	9.1	4	28.6	0.18
Dysarthria	3	13.6	0	0	0.08

*General neurological and surgical complications*					
Mania/hypomania	2	9.1	1	7.1	0.84
Perioperative confusion	2	9.1	0	0	0.16
Weight gain	8	36.4	5	35.7	0.96
Pulmonary edema	1	4.5	1	7.1	0.74
Asymptomatic intracerebral hemorrhage	1	4.5	0	0	0.43
Symptomatic intracerebral hemorrhage	1	4.5	0	0	0.43

*Hardware-related complications*					
Lead problems	1	4.5	0	0	0.43
Leads that needed to be repositioned (unilateral)	1	4.5	0	0	0.43
Infections of the hardware	1	4.5	0	0	0.43
Required removal of the system	1	4.5	0	0	0.43
Swelling region of the IPG/extension cables	1	4.5	1	7.1	0.74
Battery failure	1	4.5	0	0	0.43
Wire revision	1	4.5	0	0	0.43

GA: general anesthesia; LA: local anesthesia; IPG: implantable pulse generator.

## Data Availability

The data used to support the findings of this study are included within the article.

## Supplementary Materials

Supplementary Materials Table. Comparison of postoperative outcomes by DBS between GA and LA groups at 1 year.
