# Corrigendum to “Five-Year Clinical Outcomes of Local versus General Anesthesia Deep Brain Stimulation for Parkinson's Disease”

**DOI:** 10.1155/2019/2654204

**Published:** 2019-11-20

**Authors:** Sheng-Tzung Tsai, Tsung-Ying Chen, Sheng-Huang Lin, Shin-Yuan Chen

**Affiliations:** ^1^Department of Neurosurgery, Tzu Chi General Hospital, Tzu Chi University, Hualien, Taiwan; ^2^Institute of Medical Sciences, Tzu Chi University, Hualien, Taiwan; ^3^Department of Anesthesiology, Tzu Chi General Hospital, Tzu Chi University, Hualien, Taiwan; ^4^Department of Neurology, Tzu Chi General Hospital, Tzu Chi University, Hualien, Taiwan

In the article titled “Five-Year Clinical Outcomes of Local versus General Anesthesia Deep Brain Stimulation for Parkinson's Disease” [1], there was an error in Methods where the IRB “no. 097-32” was missing. The sentence “The institutional review board of Tzu Chi General Hospital approved this study (no. 097-08)” should be corrected as follows: “The Institutional Review Board of Tzu Chi General Hospital approved the studies (no. 097-08 and no. 097-32).”
