# Supplementary material for: Five-Year Clinical Outcomes of Local versus General Anesthesia Deep Brain Stimulation for Parkinson's Disease
Source: Parkinsons Dis. 2019 Jan 17;2019:5676345. doi: 10.1155/2019/5676345 (PMC6360066; doi:10.1155/2019/5676345)
Supplement: Supplementary Materials — Table. Comparison of postoperative outcomes by DBS between GA and LA groups at 1 year. [file 5676345.f1.pdf]

Table. Comparison of post-operative outcomes by DBS between GA and LA groups at 1 year

	GA									LA									P-value
	Levodopa off DBS off			Levodopa off DBS on			Improvement (%)			Levodopa off DBS off			Levodopa off DBS on			Improvement (%)			
UPDRS	Mean	±	SD	Mean	±	SD	Mean	±	SD	Mean	±	SD	Mean	±	SD	Mean	±	SD	(%)
Part I	3.4	±	1.3	2.3	±	1.3	29.5	±	46.6	3.5	±	1.9	2.2	±	1.4	27.4	±	31.4	0.84
Part II	19.3	±	8.0	9.4	±	5.0	47.2	±	22.6	17.2	±	7.9	7.9	±	2.9	49.5	±	19.1	0.88
Part III	45.5	±	13.5	24.7	±	10.7	45.7	±	17.1	38.2	±	15.0	17.2	±	7.6	52.6	±	18.6	0.17
Brady	19.1	±	5.7	12.3	±	4.9	35.0	±	19.5	16.4	±	5.8	9.1	±	4.3	43.1	±	26.3	0.31
Tremor	6.9	±	4.9	2.0	±	2.6	65.8	±	37.1	5.2	±	3.8	0.7	±	0.9	82.6	±	23.4	0.14
Rigidity	9.9	±	3.6	4.3	±	3.6	60.2	±	28.7	7.8	±	4.4	2.8	±	1.9	59.2	±	28.2	0.96
Posture & Gait	3.7	±	1.5	2.2	±	1.5	43.0	±	33.5	3.3	±	1.8	1.9	±	1.3	36.7	±	35.2	0.65
Axial	8.0	±	3.2	5.4	±	2.6	29.5	±	29.6	7.4	±	4.2	4.2	±	2.2	32.4	±	29.5	0.77
Part IV	5.6	±	3.2	1.6	±	1.9	40.0	±	95.5	4.7	±	3.3	1.2	±	1.7	37.2	±	92.9	0.89
Total	73.8	±	23.0	38.0	±	15.4	47.3	±	16.4	63.6	±	26.1	28.5	±	11.3	52.1	±	15.9	0.48
H & Y stage	2.9	±	0.8	2.5	±	0.7	13.7	±	20.0	2.8	±	0.6	2.2	±	0.5	19.6	±	16.8	0.36
ADL score	80.0	±	16.2	93.5	±	8.8	14.6	±	14.5	80.0	±	15.3	96.9	±	4.8	17.4	±	15.5	0.65

UPDRS: Unified Parkinson’s Disease Rating Scale, H&Y: Hoehn and Yahr, ADL: activities of daily living, GA: general anesthesia, LA: local anesthesia
